# A Simple Assay to Assess *Salmonella enterica* Persistence in Lettuce Leaves After Low Inoculation Dose

**DOI:** 10.3389/fmicb.2020.01516

**Published:** 2020-07-14

**Authors:** Paula Rodrigues Oblessuc, Maeli Melotto

**Affiliations:** Department of Plant Sciences, University of California, Davis, Davis, CA, United States

**Keywords:** lettuce, *Salmonella enterica*, apoplastic persistence, vacuum inoculation, bacterial population growth, fresh produce safety, lettuce cultivars

## Abstract

*Salmonella enterica* is an enterobacterium associated with numerous foodborne illnesses worldwide. Leafy greens have been a common vehicle for disease outbreaks caused by *S. enterica*. This human pathogen can be introduced into crop fields and potentially contaminate fresh produce. Several studies have shown that *S. enterica* can survive for long periods in the plant tissues. Often, *S. enterica* population does not reach high titers in leaves; however, it is still relevant for food safety due to the low infective dose of the pathogen. Thus, laboratory procedures to study the survival of *S. enterica* in fresh vegetables should be adjusted accordingly. Here, we describe a protocol to assess the population dynamics of *S. enterica* serovar Typhimurium 14028s in the leaf apoplast of three cultivars of lettuce (*Lactuca sativa* L.). By comparing a range of inoculum concentrations, we showed that vacuum infiltration of a bacterium inoculum level in the range of 3.4 Log CFU ml^–1^ (with a recovery of approximately 170 cells per gram of fresh leaves 2 h post inoculation) allows for a robust assessment of bacterial persistence in three lettuce cultivars using serial dilution plating and qPCR methods. We anticipate that this method can be applied to other leaf–human pathogen combinations in an attempt to standardize the procedure for future efforts to screen for plant phenotypic variability, which is useful for breeding programs.

## Introduction

Fruits and vegetables are known to have high nutrient content, making them the basis of a healthy diet. Many of these foods can be eaten raw, and although this represents a practical advantage, it also makes them notoriously relevant to foodborne illnesses. *Salmonella enterica* is one of the most common human pathogens found in fresh produce (Bennett et al., 2018; Melotto et al., 2020). Previously, plants were thought to be passive vectors for human pathogens, but recent studies showed that *S. enterica* can induce plant defense responses (Meng et al., 2013; Garcia and Hirt, 2014; Melotto et al., 2014; Oblessuc et al., 2020). Intriguingly, although the mechanism is not fully understood, this bacterium can overcome plant defense (Roy et al., 2013; Wahlig et al., 2019) and survive for weeks inside diverse plants species, including lettuce (*Lactuca sativa* L.) (Islam et al., 2004; Kroupitski et al., 2009, 2011; Jechalke et al., 2019; Roy and Melotto, 2019). These findings have prompted further research on the interaction between plants and human pathogens.

Artificial inoculation of plants is a common technique used to study plant interaction with phytopathogens (Katagiri et al., 2002; Jacob et al., 2017). Nevertheless, this approach has some technical limitations when studying plant interaction with enterobacteria, in particular *S. enterica* and enterohemorrhagic *Escherichia coli*, due to the relative low number of these bacteria inside the plant. In fact, recent studies have shown that *S. enterica* population can decrease with time in many plant species in an inoculum concentration-dependent manner (Deblais et al., 2019; Jechalke et al., 2019). Beyond that, the plant species and the inoculation procedure itself can affect bacterial population dynamics inside plants. For instance, tomato (*Solanum lycopersicum*) seedlings dip-inoculated with *S. enterica* at a concentration of 8 Log CFU ml^–1^ maintains the population size 1 day after inoculation (DAI) followed by a decrease after 14 DAI (Barak et al., 2011). Similarly, when adult lettuce leaves were dip-inoculated with 8 Log CFU ml^–1^ of *S. enterica*, the Log CFU cm^–2^ of leaf showed no alteration in bacterial population until three DAI, but a reduction in the population size after 7 DAI (Roy and Melotto, 2019). Nonetheless, when lower inoculum concentration of 4.7 Log CFU ml^–1^ of *S. enterica* was used to infiltrate small areas of fully expanded *Nicotiana benthamiana* leaves, a 100-fold increase in bacterial population was observed at three DAI (Meng et al., 2013). These findings indicate that the inoculation method and/or the initial concentration of the inoculum can influence the bacterial population dynamic in leaves.

In the field, plants can be exposed to variable amounts of pathogen load depending on the source of the inoculum. In a survey to quantify *Salmonella* in irrigation water, Antaki et al. (2016) found an average of 0.03 MPN (most probable number) of cells per 100 ml of water. Additionally, animals are reservoirs of bacterial pathogens of humans and might shed high level of inoculum in their feces. For instance, cattle feces can shed *E. coli* O157 at concentrations >4 Log CFU g^–1^ (Omisakin et al., 2003), whereas some animals such as mice are considered super-shedders of *S.* Typhimurium (Gopinath et al., 2012).

Once crops are exposed to these environmental inocula, bacterial cells can internalize into edible leaves through natural openings and wounds (Brandl, 2008; Kroupitski et al., 2009; Roy et al., 2013). Understanding human pathogen survival inside the leaf apoplast is very important as this niche protects the bacterium from common sanitation procedures of leafy vegetables (Pezzuto et al., 2016), posing a risk to reach the human host. Thus, we performed vacuum infiltration procedures using a range of low to high concentrations of bacterial inoculum (3–7 Log CFU ml^–1^) to assess the effect of inoculation dose on bacterial survival and the detection limit of our procedure using contrasting lettuce cultivars over a period of 20 days. The findings of this study will assist with designing of plant phenotypic screening useful for breeding programs.

## Materials and Methods

### Plant Material and Growth Conditions

**FIGURE 1:**
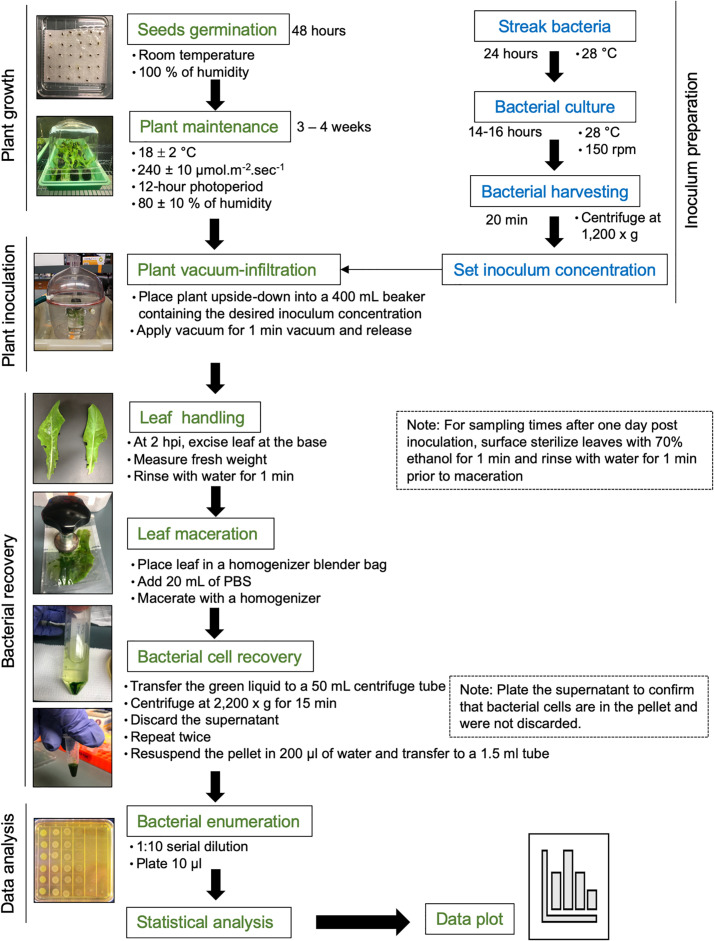
Flowchart of the procedure. Please see the section “Materials and Methods” for a detailed description.

Approximately 15 lettuce seeds of each cultivar (Red Tide, Lollo Rossa, and Salinas) were germinated in water-soaked filter paper for 2 days at room temperature. Each germinated seed was transplanted to a peat pot pre-soaked with distilled water for 10–20 min. Ideally, sprouted seeds with approximately the same root size should be selected for transplanting. Pots were placed in trays covered with plastic dome, leaving a small space (around 4 cm) to avoid water condensation, and kept at 18 ± 2°C, 240 ± 10 μmol m^–2^ s^–1^ with a 12-h photoperiod, and 80 ± 10% of air relative humidity. One week after transplanting, seedlings were fertilized with 0.05 g of fertilizer per plant mixed with 30 ml of distilled water. Three- to 4-week-old plants were used for inoculation (Figure 1).

### Bacterial Inoculum Preparation

*S. enterica* subsp. *enterica* serovar Typhimurium strain 14028s was streaked from frozen glycerol culture stock on low-salt Luria Bertani (LSLB) agar plate, supplemented with 60 μg ml^–1^ kanamycin, and incubated overnight at 28°C. Late in the afternoon of the day before the inoculation assay (around 5 pm), one single colony was placed in 100 ml of LSLB medium with 60 μg ml^–1^ kanamycin in a 125-ml Erlenmeyer flask. As a blank control, 5 ml of the LSLB plus antibiotic solution was placed into a clean culture tube. Bacterial and blank solutions were incubated in a rotary shaker at 28°C, 150 rpm, overnight (Figure 1).

In the morning of the next day, bacterial and blank solutions were removed from the incubator and the optical density at 600-nm wavelength (OD_600_) was measured using a spectrophotometer. It is important to shake the culture flask before transferring 1 ml to a sterile cuvette to avoid errors during OD readings due to bacterial settling on the bottom of the flask. The OD_600_ should be between 0.8 and 1.0 to ensure that the bacterial growth is still in the log phase. A two-step bacterial dilution was used to prepare the final inoculum at the desired concentration. Step 1: the volume of the bacterial solution needed to obtain a bacterial OD_600_ of 0.2 was calculated using the formula C1 × V1 = C2 × V2, where C = concentration and V = volume. After transferring the desired bacterial solution volume (V2) to a 50-ml centrifuge tube, bacterial cells were harvested by centrifugation at 1,200 × *g* for 20 min at 22 ± 2°C. The supernatant was discarded, and the pellet was resuspended in sterile distilled water by vortexing. The centrifugation step was essential to remove the excess of LBLS media plus kanamycin, to avoid bacterial growth inhibition within the leaf due to the presence of the antibiotic, as well as to reduce the volume of *Salmonella* solution handled in the lab. Step 2: 0.0001, 0.01, 1, or 100 ml of the final solution from step 1 (OD_600_ = 0.2) was added to a new flask containing 1,000 ml of sterile distilled water to obtain the final inoculum concentration of OD_600_ 0.00000002 (1 Log CFU ml^–1^), 0.000002 (3 Log CFU ml^–1^), 0.0002 (5 Log CFU ml^–1^), or 0.02 (7 Log CFU ml^–1^). Finally, 0.1 ml of Silwet was added to the inoculum to obtain a final concentration of 0.01%. Inoculum was stirred with a magnetic bar.

### Vacuum Inoculation of Lettuce Leaves

Three- to four-week-old lettuce plants (four plants per cultivar) were vacuum-infiltrated with the final bacterial solution of 1.8, 3.5, 5.4, or 7.7 Log CFU ml^–1^. These concentration values were estimated by serial dilution plating of the inoculum. Each potted plant was placed upside-down into a 400-ml beaker containing enough inoculum to immerse the plant shoot completely. Aluminum foil was placed at the base of the plant to avoid the contact of soil with the inoculum. Submerged plants were placed in a vacuum chamber and vacuum was applied for 1 min. To enable a uniform filling of the leaf apoplast with inoculum, the vacuum was released quickly by disconnecting the suction tube to the vacuum chamber, allowing the chamber to depressurize. The leaves should become dark green due to inoculum infiltration (Figure 1). Fresh inoculum was added to the beaker to ensure total immersion of the inoculated leaves and after three plants were inoculated. Inoculated plants were placed back in the trays and partially covered with the plastic dome for the duration of the experiment.

### Enumeration of Apoplastic Bacterium Population

Bacterial population size was estimated in the second true leaf of the inoculated plants at 2 h post inoculation (HPI), and 1, 10, and 20 DAI. After 2 HPI when the water soaking disappeared from the leaves, the second true leaf was excised at its base, the fresh weight (FW) was measured using an analytical balance, and the leaf was rinsed by immersion in sterile distilled water for 1 min. Sampling at this time point was crucial to determine the total number of bacteria inoculate on and in the plant. For the following time points, after FW measurement, the leaf was surface sterilized with 70% ethanol for 1 min and rinsed in water for 1 min.

For all time points, the excess water was removed from the leaves by gently blotting them on paper towel. Leaf was placed in a homogenizer blender bag containing 20 ml of phosphate-buffered saline (PBS) solution and macerated with a homogenizer until a green solution with very small leaf debris was obtained. The green solution, but not the leaf debris, was transferred to a 50-ml centrifuge tube and centrifuged at 2,200 × *g* for 15 min at 22 ± 2°C. The supernatant was removed, and fresh 20-ml PBS solution was added to the pellet containing bacterial cells, followed by centrifugation at 2,200 × *g* for 15 min (Figure 1). To confirm that no bacterium was present in the supernatant, this solution was plated on solid LSLB medium.

After the wash-centrifugation steps, 0.2 ml of sterile Milli-Q water was added to recover the bacterial pellet and transferred to a clean 1.7-ml tube. Please note that water was used as further DNA extraction was desired, but PBS buffer could be used if only serial dilution and plating would be performed. Immediately after bacterial recovery, 10 μl of the bacterial solution was added to 90 μl sterile water in another 1.7-ml microfuge tube, making a 1:10 dilution. This solution was diluted to 10^–2^ for the low inoculum dose and down to 10^–8^ for the highest inoculation dose. Including the most concentrated leaf sample, 10 μl of all dilutions was plated on LBLS agar with 60 μg ml^–1^ kanamycin (Figure 1; Jacob et al., 2017). Dilution plates were air-dried and subsequently incubated at 28°C overnight. The next day, bacterial colonies were counted at the dilution column that allowed for the visualization of individual colonies using a stereoscope.

### Data Analysis

The number of single colony-forming units (CFU) was used to estimate the bacterial population per gram of fresh leaf tissue by multiplying the CFU counts by the dilution factor times 10, to account for the 10 μl out of the 100 μl used for plating. Data points represent the average of three biological replicates (three different plants) and two technical replicates during plating (*n* = 6). Average and the standard error (SE) were calculated using Microsoft Excel. Statistical significance among the different cultivars and time points was estimated by the analysis of variance (ANOVA) followed by Scott–Knott test with a significance threshold of α = 0.05, using the square root of the means. This data transformation method is recommended when the variance is proportional to the mean (Manikandan, 2010). The graph was plotted with the Log CFU per gram of leaf FW over time using untransformed data.

### Total DNA Extraction and qPCR Analysis

After the wash-centrifugation steps described above, 5 μl of the DNA extraction buffer was added to 50 μl of the recovered bacterial solution. After vortexing for 30 s, the solution was centrifuged for 1 min at 13,000 rpm at room temperature, and the supernatant was transferred to a clean tube. DNA in the supernatant was precipitated by adding 0.1 volumes of 5 M ammonium acetate and one volume of isopropanol, followed by vortexing and 1-h incubation at room temperature. Next, two washes were performed to remove excess salt by adding 1 ml of cold 70% ethanol, vortexing for 30 s, and spinning-down for 1 min at 13,000 rpm, room temperature. After each centrifugation, the ethanol solution was discarded. Finally, the DNA pellet was dried out on the bench for 15 min and resuspended in 30 μl of DNase-free water. Quantitative PCR (qPCR) was performed with 3 ng of DNA template, 200 nM of reverse and forward gene-specific primers, and 10 μl of iTaq Fast SYBR Green Supermix in a total reaction volume of 20 μl. Reactions were carried out in an Applied Biosystems 7300 thermocycler, using the following cycling parameter: 1 cycle of 95°C for 5 min, and 40 cycles of 95°C for 10 s and 60°C for 30 s. The dissociation curve was determined for every reaction to confirm the presence of a single amplicon and the lack of primer dimers and non-specific products.

The primer set efficiency was assessed using the standard curve method. The linear regression equation was plotted using the cycle threshold (CT) value and the Log of the DNA concentrations of 10-fold serial dilutions, using the Microsoft Excel software. The slope values were used to calculate the efficiency for each pair of primers tested (Kralik and Ricchi, 2017) and number of DNA copies (Brankatschk et al., 2012). *S. enterica*-specific primers (forward—TCGTCATTCCATTACCTACC and reverse—AAACGTTGAAAAACTGAGGA; Hoorfar et al., 2000) and the ribosomal 16S primers (forward—CCAGCAGCCGCGGTAAT and reverse—TTTACGCCCAGTAATTCCGATT; Choi et al., 2017) were selected for this assay. The number of DNA copies per gram of leaf tissue was calculated using the formula: number of DNA copies = (ng × 6.002 × 10^23^)/(length × 1 × 10^9^ × 650), in which ng is the Log (CT – standard curve intercept/slope standard curve), 6.002 × 10^23^ is Avogadro’s number, length is the size of the *S. enterica* strain 14028s genome (4,964,097 bases), 1 × 10^9^ is used to account for the ng unit conversion, and 650 is the molar mass in grams per mole of one single DNA base pair (Brankatschk et al., 2012). The number of DNA copies is equal to the number of cells per reaction for 1 μl of DNA sample, when using *S. enterica*-specific primers.

### List of Materials

• Peat Pellets 42 mm (peat moss pellets) (Jiffy 7, catalog number: SO-JFPP).
• Plastic trays without holes (Hummert International, catalog number: 65-6963-2).
• Fertilizer (Peters Excel^®^ pHLow^®^ 19-11-21 Multi-Purpose, catalog number: G99001).
• Plastic domes (Hummert International, catalog number: 65-6964-1).
• Soil mix (Sun Gro^®^ Sunshine^®^ #1 Grower Mix with RESILIENCE^TM^).
• 50-ml centrifuge tubes (Fisher Scientific, catalog number: 553860).
• 1.7-ml microcentrifuge tubes (VWR, catalog number: 87003-294).
• Culture Tubes, Plastic, with Dual-Position Caps (VWR, catalog number: 60818-703).
• 125-ml Erlenmeyer flasks (Pyrex^®^, catalog number: 4980-125).
• 250-ml and 1,000-ml beakers (VWR, catalog numbers: 10754-952 and 10754-960).
• Filter Whirl-Pak(R) homogenizer blender filter bag 207 ml (Millipore Sigma, catalog number: WPB01385WA-250EA).
• Sterile inoculating loops (VWR, catalog number: 82051-146).
• Magnetic stir bars (VWR, catalog number: 58948-988).
• Square petri dish with grid (VWR, catalog number: 60872-310).
• Round petri dishes, medium (100 × 15 mm) (VWR, catalog number: 25384-302).
• Disposable plastic cuvettes (VWR, catalog number: 97000-586).
• Micropipettes (Rainin Pipet-Life^TM^).
• Tweezers (VWR, catalog number: 89259-984).
• Silwet L-77 (Lehle Seeds, catalog number: VIS-30).
• Agarose (VWR, catalog number: 97062-250).
• Tryptone (IBI Scientific, catalog number: 41116105).
• Yeast extract (US Biotech Sources, catalog number: Y01PD-500).
• Sodium chloride (Fisher Scientific, catalog number: S271-500).
• Bacteriological agar (IBI Scientific, catalog number: IB49171).
• LSLB medium (broth and agar; see Recipes).
• TRIS—tris(hydroxymethyl)aminomethane (VWR, catalog number: 33621.260).
• EDTA—ethylenediaminetetraacetic acid (VWR, catalog number: 20294.294).
• SDS—sodium dodecyl sulfate (VWR, catalog number: 1.13760.0100).
• Ammonium acetate (VWR, catalog number: 0103-500G).
• Kanamycin (GoldBio, catalog number: K-120).
• iTaq Fast SYBR Green Supermix (BioRad, Hercules, CA, United States).
• Sterile distilled water.
• Sterile Milli-Q water.
• Ethanol pure grade (Sigma-Aldrich, catalog number: 459836).
• PBS buffer (see Recipes).
• Lettuce cultivars (Red Tide, Lollo Rossa and Salinas, stored at 4°C).
• *S. enterica* stock cultures (stored in 20% glycerol at −80°C).

### Required Equipment

• Plant growth chamber (Caron Products & Services, model: 6341-2).
• Shaker incubator (VWR, catalog number: 12620-946).
• Spectrophotometer (Thermo Fisher Scientific, model: Spectronic 20D + or equivalent).
• Centrifuge (Eppendorf, model: 5810).
• Homogenizer Hand Model (Bioreba, catalog number: 400010).
• Digital hygrometer (VWR, catalog number: 35519-047).
• Quantum meter (Apogee, catalog number: BQM).
• Vortex (BioExpress, GeneMate, catalog number: S-3200-1).
• Analytical Balance (VWR, catalog number: 10753-570).
• Magnetic stirrer (VWR, catalog number: 97042-642).
• Stereoscope (VWR, catalog number: 89404-502).
• Applied Biosystems 7300 thermocycler (Applied Biosystems, Foster City, CA, United States).
• 20-, 200-, and 1,000-μl micropipettes and tips.
• Milli-Q filter (Millipore Sigma, catalog number: C85358).
• Autoclave.
• Biological safety cabinet level 2 (Labconco^TM^ Purifier^TM^ Axiom^TM^ Class II, Type C1, Kansas City, MO, United States).

### Solution Recipes

#### Low-Sodium Luria Bertani Medium

10 g L^–1^ Tryptone
5 g L^–1^ Yeast extract
5 g L^–1^ NaCl
15 g L^–1^ Agar (only for solid medium)
Autoclave medium at 15 psi, 120°C for 15 min.
Allow medium to cool down to about 55°C and add appropriate antibiotic if needed.

#### Phosphate-Buffered Saline Solution

8 g L^–1^ NaCl
0.2 g L^–1^ KCl
1.44 g L^–1^ Na_2_HPO_4_
0.24 g L^–1^ KH_2_PO_4_

#### DNA Extraction Buffer

200 mM Tris (pH 7.5)
250 mM NaCl
25 mM EDTA
0.5% SDS

## Results

Previously, it was reported that *S. enterica* persistence is dependent on the bacterial inoculum concentration (Deblais et al., 2019; Jechalke et al., 2019) and on the lettuce cultivar (Jacob and Melotto, 2020). Therefore, we tested whether our protocol was useful to reliably enumerate bacterial cells in lettuce leaves using four different concentrations of bacterial inoculum (1, 3, 5, or 7 Log CFU ml^–1^) and three commercial cultivars of lettuce with contrasting bacterial growth patterns (Red Tide, Lollo Rossa, and Salinas). The lowest inoculum concentration (1 Log CFU ml^–1^) is impractical to use as no live bacteria could be recovered at 2 HPI, i.e., no colonies grew on the medium after plating.

**FIGURE 2:**
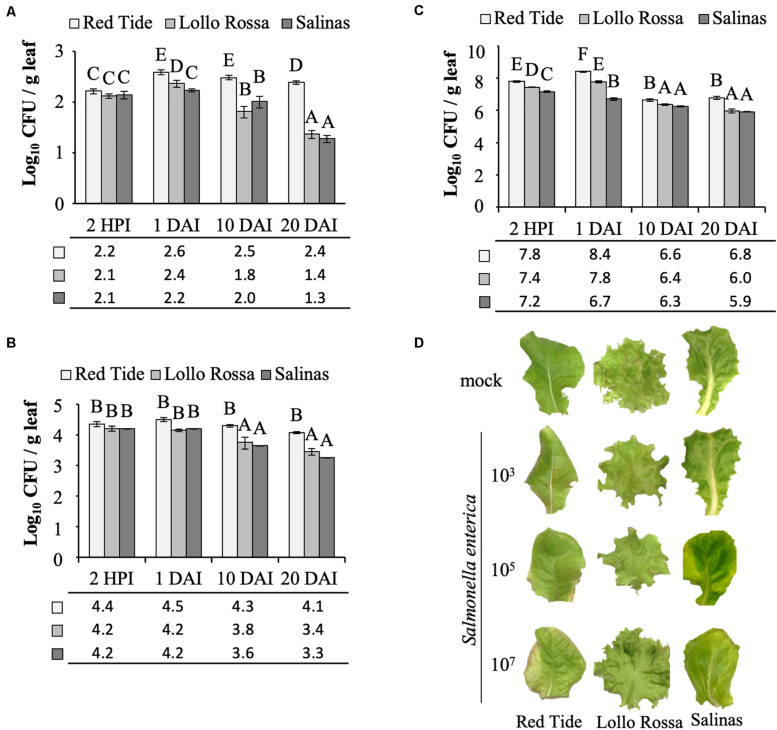
*Salmonella enterica* persistence in the apoplast of lettuce leaves. Bacterial population size was estimated at 2 h post inoculation (HPI), and at 1, 10, and 20 days after vacuum inoculation (DAI) with the strain 14028s of *S. enterica* subsp. *enterica* serovar Typhimurium at a concentration of **(A)** 3.5 Log CFU ml^–1^, **(B)** 5.4 Log CFU ml^–1^, or **(C)** 7.7 Log CFU ml^–1^. Results are shown as the average of three biological replicates and two technical replicates (*n* = 6 ± SE). Statistical difference among means was detected with ANOVA followed by the Scoot–Knott test (α = 0.05). Different letters above the bars indicate significant statistical differences among cultivars across all time points. CFU = colony forming unit. g, grams. **(D)** Representative pictures of lettuce leaves at 20 DAI with each one of the bacterial inoculum dose used (3, 5, or 7 Log CFU ml^–1^) or with the mock control.

At the inoculum concentration of 3 Log CFU ml^–1^, *S. enterica* grew in Red Tide leaves with a 2.3-fold increase in CFU observed between 2 HPI and 1 DAI, while in Lollo Rossa and Salinas, the bacterial titer showed a 1.8-fold or no increase in the same time period (*p* < 0.05), respectively (Figure 2A). From 1 to 20 DAI, the bacterial population decreased for all plant cultivars. However, the extent of bacterial population decrease was smaller in Red Tide (1.6-fold), whereas it decreased 10-fold in Lollo Rossa and 8.9-fold in Salinas (*p* < 0.05; Figure 2A).

When inoculated with 5 Log CFU ml^–1^, a similar trend of higher bacterial population in Red Tide leaves as compared to Lollo Rosa and Salinas was observed throughout the experiment (*p* < 0.05). However, the bacterium CFU per gram of leaf remained constant in Red Tide until 20 DAI (Figure 2B). Bacterial population inside Lollo Rossa and Salinas leaves remained constant between 2 HPI and 1 DAI; however, the bacterial titers decreased 2.4- and 3.6-fold between 1 and 10 DAI in Lollo Rossa and Salinas, respectively (*p* < 0.05). By 20 DAI, *S. enterica* decreased further in Lollo Rossa and Salinas by 5.0- and 8.9-fold, respectively (*p* < 0.05; Figure 2B).

Remarkably, at 2 HPI with 7 Log CFU ml^–1^ of *S. enterica*, a significantly larger bacterial population in Red Tide leaves was observed in comparison to that in Lollo Rossa and Salinas (Figure 2C). The bacterial population further increased by 4.1-fold at 1 DAI in Red Tide leaves, but subsequently decreased by 56.8- and 40.9-fold at 10 and 20 DAI, respectively (*p* < 0.05; Figure 2C). Lollo Rossa also supported a higher number of *S. enterica* cells at 1 DAI, a 2.3-fold increase from 2 HPI, followed by a decrease of 26.5- and 64.9-fold between 1 and 10 DAI or 1 and 20 DAI, respectively (*p* < 0.05; Figure 2C). Bacterial growth inside Salinas leaves had a 2.9-fold decrease in population size between 2 HPI and 1 DAI, also decreasing the number of bacterial populations in its leaves by 2.8- and 6.3-fold at 10 and 20 DAI, respectively (*p* < 0.05; Figure 2C). These findings suggest that high levels of inoculum concentration lead to a higher *S. enterica* death rate inside the leaf apoplast, independently of the plant cultivar, considering that the second true leaf was fully expanded before inoculation and the role leaf was sampled. Although Red Tide supported larger bacterial populations at all times (Figure 2C).

Overall, our results showed that, independently of the inoculation dose, Red Tide supported higher bacterial population than Lollo Rossa and Salinas, in which the inoculum concentration of 3 Log CFU ml^–1^ enhanced these differences, mainly at later time points (Figure 2). No macroscopic symptoms such as chlorosis or necrosis were observed on mock-inoculated leaves or leaves inoculated with 3 Log CFU ml^–1^ of *S. enterica*, for all three cultivars tested. Red tide showed some chlorosis when inoculated with 5 and 7 Log CFU ml^–1^, while no chlorosis was observed for Lollo Rossa or Salinas when these same concentrations of bacterial inoculum were used (Figure 2D).

**FIGURE 3:**
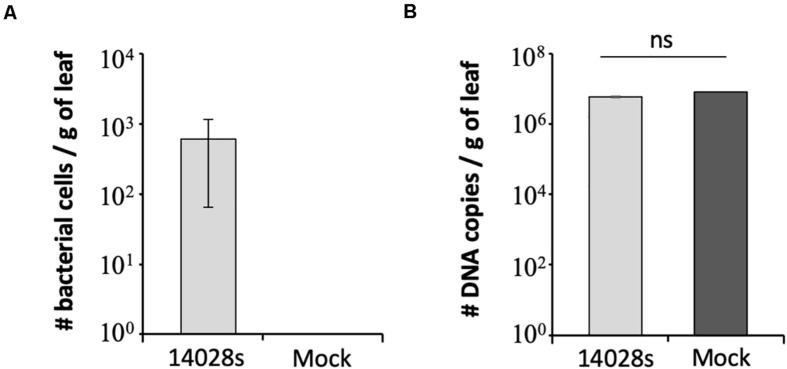
Number of DNA copies estimated by qPCR with **(A)** *Salmonella enterica*-specific primers (Hoorfar et al., 2000) or **(B)** 16S primers (Choi et al., 2017) in Red Tide leaves. Bacterial population (*S. enterica* strain 14028s) was determined at 1 day post vacuum inoculation with 3.5 Log CFU ml^–1^ of bacterial cells or water + 0.01% Silwet as a mock control. Results are shown as the average of three biological replicates (*n* = 3 ± SE). Value of the mock treatment in graph **(A)** is zero and the error bars are very small to appear in graph **(B)**. CFU = colony forming unit, g, gram; ns, non-significant statistically.

To support the results of the serial dilution plating method in estimating the bacterial population size, we used qPCR as a second approach (Fu et al., 2006). This is a simple assay widely used in microbial community analysis, as well as it is quick and less labor-intensive than other methods (Brankatschk et al., 2012; Davis, 2014). To this end, we chose to test the sample from Red Tide leaves at 1 day post vacuum inoculation with 3 Log CFU ml^–1^ bacteria. Due to the small number of recovered bacterial cells (∼390 ± 41.6 cells per gram of leaf) (Figure 2A), technical error during plating could have occurred. Using the *S. enterica*-specific primers, we estimated that 612 ± 54.7 bacterial cells were present per gram of leaf, while no amplicon was detected in mock-inoculated leaves, the negative control (Figure 3A). To rule out the possibility that the lack of amplicon was due to the lack of DNA in the PCR reaction, the 16S primer set was used with the same DNA samples from bacterium- and mock-inoculated leaves. This primer set also aligns with the lettuce mitochondrion genome (NCBI reference NC_042756.1, e-value between 1 × 10^–3^ and 6 × 10^–4^); thus, amplification of both plant and bacterium DNA was expected. Similar amount of DNA was recovered from both *S. enterica*- and mock-inoculated plants, indicating a consistent DNA extraction protocol, in which DNA from bacteria was precipitated together with the plant DNA (Figure 3B).

## Discussion

Although *S. enterica* induces plant defense responses (Meng et al., 2013; Garcia and Hirt, 2014; Melotto et al., 2014; Oblessuc et al., 2020), it can still persist for long periods in the leaf apoplast depending on the bacterial strain and the plant genotype (Wong et al., 2019; Jacob and Melotto, 2020). Furthermore, recent studies have shown that variations in the *S. enterica* culturing conditions, such as temperature and nutrients in the medium (Kroupitski et al., 2019), and environmental conditions for the plant cultivation, such as temperature and humidity (Deblais et al., 2019; Jechalke et al., 2019; Roy and Melotto, 2019), can interfere mainly with the ability of *S. enterica* to internalize plant tissues. Nevertheless, variations in environmental conditions not only affect the bacterial internalization, which indeed is an important step during bacterial colonization of plants, but also interfere with the outcome of the plant–pathogen interactions and the persistence phenotype. These findings highlight the importance of establishing inoculation procedures and bacterial enumeration methods with controlled conditions that enable a realistic understanding of *S. enterica* survival in the plant, isolating the plant phenotype from environmental effects.

The method described here is simple and robust to assess *S. enterica* persistence in plant leaf apoplast and, in addition, to allow for comparisons among different inoculation doses and lettuce cultivars. We have determined that 3 Log CFU ml^–1^ is the minimum concentration of bacterial inoculum in which bacterial cells can be reliably recovered from leaves right after inoculation. This inoculum level also enabled us to follow the drastic decrease of bacterial population size in the cultivars Lollo Rossa and Salinas, when 23 ± 3.2 and 19 ± 3.2 cells per gram of leaf was detected at 20 DAI, respectively (Figure 2A). We used two methods to verify the results, in which the number of bacterial cells per gram of leaf tissue estimated by plating or qPCR was comparable. The plating method estimated the number of live bacterial cells only, whereas qPCR amplifies DNA from all cells present in the tissue, which might explain the lower cell number estimate by plating (390 ± 41.6 cells) as compared to qPCR (612 ± 54.7 cells). Plating also has the advantage of being cheaper and less labor-intensive than qPCR. However, if automation is an option, qPCR might be a better choice for larger screening procedure.

In addition to bacterial plating and qPCR techniques, other methods are known to be used to enumerate bacterial cells. Among these, treatment with propidium monoazide (PMA) alone or PMA + deoxycholate (DC) can be used before DNA extraction to detect bacterial cell death in the qPCR analysis. However, it may kill cells injured from experimental treatments that otherwise could have recovered (Laidlaw et al., 2019). Moreover, microscopy techniques, such as fluorescent *in situ* hybridization (FISH), and cell sorting techniques, such as flow cytometry (FC) and the specialized method of fluorescence-activated cell sorting (FACS), can also be used to access bacterial population (Davis, 2014), but these are expensive and labor-intensive. Hence, frequently conventical plating is qualified as the most robust and reliable method for cell quantification (Brankatschk et al., 2012; Laidlaw et al., 2019).

Understanding the various aspects of human bacterial pathogen interactions with plants is important to establish successful strategies to prevent, or at least reduce, contamination of fresh produce. We anticipate that this method will enable one to address questions related to the survival of human pathogens in leaves, such as the plant immune responses triggered by them, how human pathogens can affect the plant environment and its microbial community, and the mechanisms involved in the process. It is important to note that we chose vacuum infiltration in order to address bacterial survival in the leaf apoplast, but if internalization processes are the goal of future studies, we would indicate dip or spray inoculation followed by leaf surface sterilizing and print of the leaves in a petri dish with culture media, to confirm that the sterilization procedure was efficient. Ultimately, this procedure can be used to phenotype mapping populations to further identify genomic regions in the plant associated with defense against *S. enterica*, in addition to be useful for bacterial competition assays *in planta* to determine the relative fitness of various strains in this niche.

## Data Availability Statement

All datasets generated for this study are included in the article/supplementary material.

## Author Contributions

PO and MM conceived the research, analyzed the data, and wrote the manuscript. PO performed the experiments. Both authors contributed to the article and approved the submitted version.

## Conflict of Interest

The authors declare that the research was conducted in the absence of any commercial or financial relationships that could be construed as a potential conflict of interest.
